# Metagenomic Features Characterized with Microbial Iron Oxidoreduction and Mineral Interaction in Southwest Indian Ridge

**DOI:** 10.1128/spectrum.00614-22

**Published:** 2022-10-26

**Authors:** Ying-Wen Zhong, Peng Zhou, Hong Cheng, Ya-Dong Zhou, Jie Pan, Lin Xu, Meng Li, Chun-Hui Tao, Yue-Hong Wu, Xue-Wei Xu

**Affiliations:** a School of Oceanography, Shanghai Jiao Tong University, Shanghai, PR China; b Key Laboratory of Marine Ecosystem Dynamics, Ministry of Natural Resources & Second Institute of Oceanography, Ministry of Natural Resources, Hangzhou, PR China; c Archaeal Biology Center, Institute for Advanced Study, Shenzhen University, Shenzhen, Guangdong, PR China; d Shenzhen Key Laboratory of Marine Microbiome Engineering, Institute for Advanced Study, Shenzhen University, Shenzhen, Guangdong, PR China; e College of Life Sciences and Medicine, Zhejiang Sci-Tech University, Hangzhou, PR China; f Key Laboratory of Submarine Geosciences, Ministry of Natural Resources & Second Institute of Oceanography, Ministry of Natural Resources, Hangzhou, PR China; University of Minnesota

**Keywords:** Southwest Indian Ridge, microbial community, hydrothermal vent, iron oxidation, sulfur oxidation, pyrrhotite

## Abstract

The Southwest Indian Ridge (SWIR) is one of the typical representatives of deep-sea ultraslow-spreading ridges, and has increasingly become a hot spot of studying subsurface geological activities and deep-sea mining management. However, the understanding of microbial activities is still limited on active hydrothermal vent chimneys in SWIR. In this study, samples from an active black smoker and a diffuse vent located in the Longqi hydrothermal region were collected for deep metagenomic sequencing, which yielded approximately 290 GB clean data and 295 mid-to-high-quality metagenome-assembled genomes (MAGs). Sulfur oxidation conducted by a variety of Gammaproteobacteria, Alphaproteobacteria, and Campylobacterota was presumed to be the major energy source for chemosynthesis in Longqi hydrothermal vents. Diverse iron-related microorganisms were recovered, including iron-oxidizing Zetaproteobacteria, iron-reducing *Deferrisoma*, and magnetotactic bacterium. Twenty-two bacterial MAGs from 12 uncultured phyla harbored iron oxidase Cyc2 homologs and enzymes for organic carbon degradation, indicated novel chemolithoheterotrophic iron-oxidizing bacteria that affected iron biogeochemistry in hydrothermal vents. Meanwhile, potential interactions between microbial communities and chimney minerals were emphasized as enriched metabolic potential of siderophore transportation, and extracellular electron transfer functioned by multi-heme proteins was discovered. Composition of chimney minerals probably affected microbial iron metabolic potential, as pyrrhotite might provide more available iron for microbial communities. Collectively, this study provides novel insights into microbial activities and potential mineral-microorganism interactions in hydrothermal vents.

**IMPORTANCE** Microbial activities and interactions with minerals and venting fluid in active hydrothermal vents remain unclear in the ultraslow-spreading SWIR (Southwest Indian Ridge). Understanding about how minerals influence microbial metabolism is currently limited given the obstacles in cultivating microorganisms with sulfur or iron oxidoreduction functions. Here, comprehensive descriptions on microbial composition and metabolic profile on 2 hydrothermal vents in SWIR were obtained based on cultivation-free metagenome sequencing. In particular, autotrophic sulfur oxidation supported by minerals was presumed, emphasizing the role of chimney minerals in supporting chemosynthesis. Presence of novel heterotrophic iron-oxidizing bacteria was also indicated, suggesting overlooked biogeochemical pathways directed by microorganisms that connected sulfide mineral dissolution and organic carbon degradation in hydrothermal vents. Our findings offer novel insights into microbial function and biotic interactions on minerals in ultraslow-spreading ridges.

## INTRODUCTION

Mid-ocean ridges constitute the longest deep-sea mountain chains on Earth, occupying approximately one third of the area of the global ocean. Based on their spreading rate, mid-ocean ridges can be categorized into fast (full spreading rate ≥ 60 mm yr^−1^), intermediate (20 mm yr^−1^ ≤ spreading rate < 60 mm yr^−1^), slow, and ultraslow (<20 mm yr^−1^) ridges. Notably, ultraslow ridges have been recently acknowledged as a new class of mid-ocean ridges and increasingly become the ideal model for observing subseafloor geological processes for their uniqueness in morphology and crustal characteristics (1). The Southwest Indian Ridge (SWIR) is a typical ultraslow-spreading center, with a spreading rate as low as 12-15 mm yr^−1^ (2, 3), occupying approximately 10% of the area of the global oceanic ridge system (4). It is also known as one of the most complicated mid-ocean ridge systems in the global oceans, as its hydrothermal activities are under the interactive control of multiple geological factors such as magma supply, thermal sources of magmatic activities, and tectonic settings (5). Massive deep-sea research has been performed in the SWIR, seeking novel insights in subsurface hydrothermal systems (6, 7), global biogeographic connections (8), and deep-sea mining management (5, 9, 10).

The first active hydrothermal region reported in the SWIR was Longqi, which was discovered during the Chinese DY115-19 cruise in 2007 (11). Up to now, 9 active black smokers and 5 diffuse vents have been reported in Longqi (12), with the maximum temperature reaching 381°C (13). A deep subsurface circulation system featured with a high-temperature, fluid-water mixing process was identified, and may be responsible for the formation of massive sulfide deposits (6, 14, 15) and high Fe but low pH hydrothermal fluids (6). Hydrothermal activities in Longqi have also been presumed active the last 100 thousand years up to now (16), and are continually growing at matured stages (15). Previous investigations have mainly aimed to study microbial community compositions on hydrothermal vents or plumes by means of 16S rRNA amplicon sequencing or denaturing gradient gel electrophoresis (DGGE) (17–20). Ding et al. has revealed that microbial communities in Longqi were highly diverse and comparable to other vents in global oceans (17). However, little is known about the potential microbial activities in Longqi hydrothermal vent communities. It is unclear how microbial communities interact with venting activities, seawater, and chimney minerals in the Longqi area.

Biotic iron oxidation in hydrothermal vents is currently poorly understood but is known to be actively associated with local mineral biogeochemistry and microbial energy conservation (21–25). Several autotrophic iron-oxidizing species have been reported functionally versatile and metabolically active, such as Zetaproteobacteria and Gammaproteobacteria (*Thiomicrospira*) (26–28). Conversely, up to now, heterotrophic iron-oxidizing bacteria have been rarely detected and most of them are presumed to have limited abilities of conducting iron oxidation by subsidiary abiotic processes (29, 30). Heterotrophic bacteria that can direct enzymatic iron oxidation are scarcely reported across all nature ecosystems, let alone hydrothermal vent environments (29, 31). In fact, strict requirements for growth have consistently composed obstacles in the discovery and cultivation of novel iron-oxidizing species (32). However, recent progresses associated with molecular mechanisms of microbial iron oxidation, such as the recognition of Cyc2 as an iron oxidase (33–35) and the discovery of multi-heme proteins functioning in extracellular electron transportation (36, 37), have facilitated chances of studying microbial iron oxidation through bioinformatic methods. Combined with genome-level data and qualified annotation tools (28, 38–40), identification of novel iron-oxidizing species would be feasible, as well as evaluation of the microbial interactions with chimney minerals.

To deepen the understanding of the potential microbial activities in ultraslow ridges, deep metagenomic sequencing of samples from an active black smoker (DFF12) and a diffuse vent (DFF1) in the Longqi hydrothermal region were performed. Combined with mineralogical analysis, potential reconstruction of microbiome and its interactions with environment were indicated in this study.

## RESULTS

### Venting features and mineralogical composition.

Sample Dive96 was collected from DFF12, which is an active black smoker with vigorous venting activity. Sample Dive100 was collected from the diffuse vent DFF1. In 2011, DFF1 was observed as an active black smoker venting at its early stage (41). However, by the time for sample collection in 2015, venting activity in DFF1 had dramatically declined and became a diffuse vent (12). Diverse megafauna were observed in DFF1 such as small fish, shrimp, and gastropods (12). Two vents were situating at a distance of about 370 m in Longqi hydrothermal vent region.

X-ray diffraction (XRD) examination revealed that 2 samples were with distinct mineralogical composition. Bulk sulfide mineral Dive96 was mainly composed of sphalerite (ZnS) with iron-oxyhydroxide (FeOOH) covering the surface (Fig. S1 and Table S1). Anhydrite (Ca_2_SO_4_) and pyrrhotite (Fe_1-_*_x_*S) was also detected in Dive96. Dive100 formed hollow cylinder, with iron-oxyhydroxide on its outer surface and the inner wall mainly composed of sphalerite (Fig. S1).

### Metagenomic sequencing and binning.

**FIG 1:**
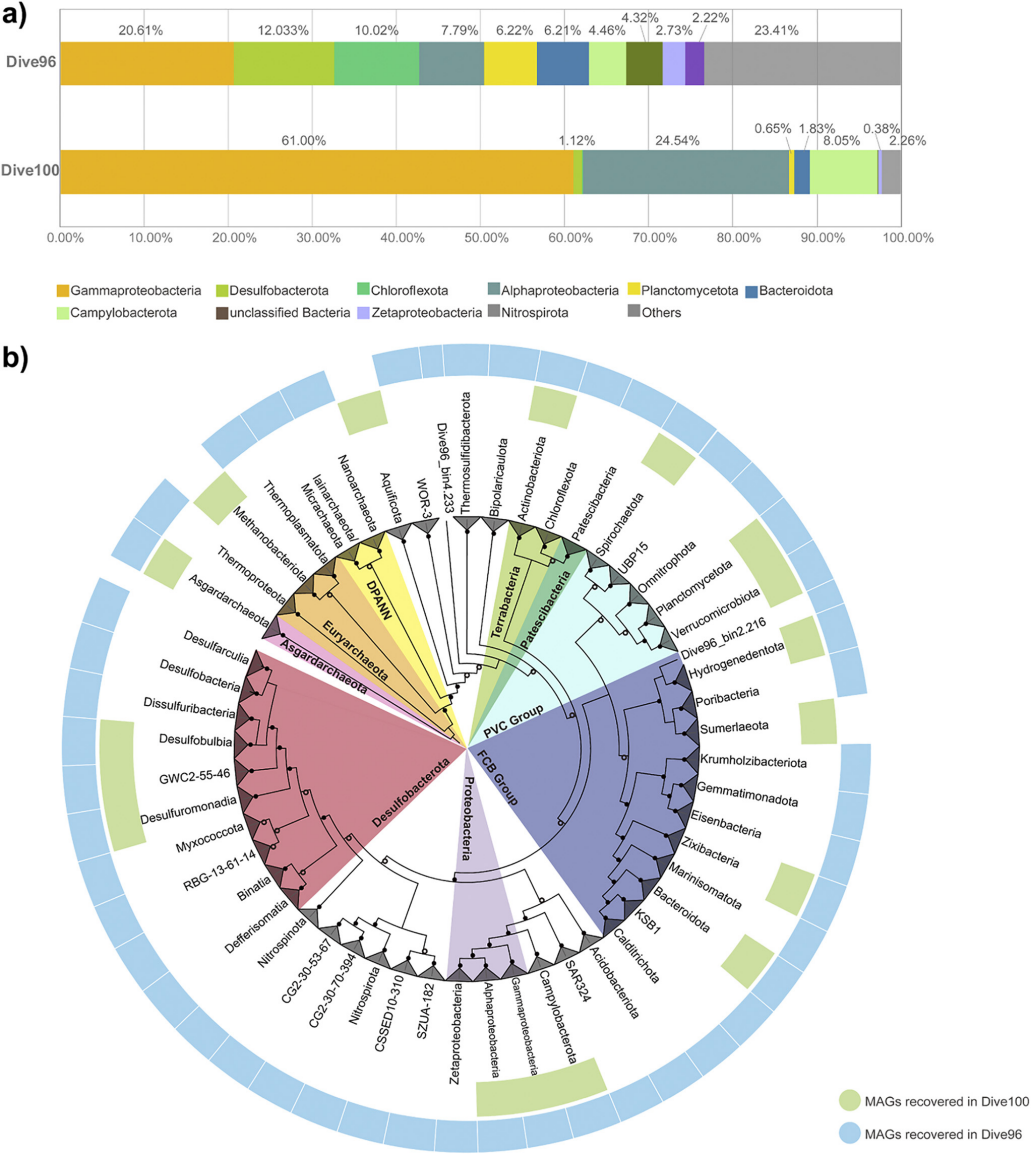
(a) Metagenomic profile of *rps3* abundance in two microbial communities. (b) Phylogeny of 295 MAGs inferred from concatenated alignment of 16 ribosomal proteins. Phylogenetic distribution of 7 archaeal phyla and 35 bacterial phyla were collapsed down to phyla or class level (Desulfobacteria). Yet MAGs Dive96_bin2.216 and Dive96_bin4.233 were presumed as unclassified bacteria due to low support in their phylogenetic positions (bootstrap values lower than 70). Solid circles inside phylogenetic tree marked nodes with bootstrap values not lower than 90. Hollow circles marked nodes with bootstrap values lower than 90 but higher than 70.

Metagenomic sequencing yielded approximately141.2 GB and 149.8 GB of clean reads for Dive96 and Dive100, respectively. The following assembly processes produced 4.6 GB and 1.1 GB contig data (Table S1). A total of 208 mid-to-high quality (completeness > 70%, contamination <10%) MAGs were recovered from Dive96, and 87 mid-to-high quality MAGs were recovered from Dive100. These MAGs represented 7 archaeal phyla and 35 existing bacterial phyla, as determined by GTDB-Tk and a phylogenetic tree constructed from concatenated alignments of 16 ribosomal proteins (Fig. 1).

### Microbial structure and composition of MAGs.

Based on an abundance profile of ribosomal protein Rps3 in the 2 metagenomes (Fig. 1), the dominant members of these microbial communities were bacteria rather than archaea. The most enriched members in these 2 bacterial communities belonged to Gammaproteobacteria, which accounted for 20.6% and 61.0% of the total Rps3 abundance in Dive96 and Dive100, respectively (Fig. 1). Campylobacterota was also detected in both samples, with relative abundance reaching 4.5% in Dive96 and 8.1% in Dive100. Chloroflexota and Desulfobacterota were enriched in Dive96, accounting for 10.0% and 12.0% of the abundance, respectively. Alphaproteobacteria were abundant in Dive100, accounting for 24.5% of the total Rps3 abundance.

Gammaproteobacteria, Alphaproteobacteria, and Desulfobacterota were also the dominant groups as each of them were represented by at least 30 MAGs in total. Common thermophilies Aquificota and *Methanocaldococcus* were detected with total MAG abundance at 0.7% in Dive100, as well as mesophilic Campylobacterota (1.9%) including *Sulfurovum*, Sulfurimonadaceae, and Sulfurospirillaceae. In Dive96, apart from Sulfurimonadaceae, thermophiles such as Thermosulfidibacterota, Dissulfuribacteria, Defferisomatia (42–45) were identified with total MAG abundance at 0.4%. Two MAGs related Nitrospirota, which was previously assumed as the indicators for recent extinct venting activities, were recovered in black smoker Dive96 with abundance at 0.5%. In addition, diverse MAGs related to uncultured FCB superphylum such as of KSB1, Krumholzibacteriota, Gemmatimonadota, Eisenbacteria, and Calditrichota were exclusively obtained in Dive96. Neither MAGs nor 16S rRNA fragments from these bacteria were detected in Dive100 (Fig. 1).

### Microbial sulfur metabolic features and associated groups.

**FIG 2:**
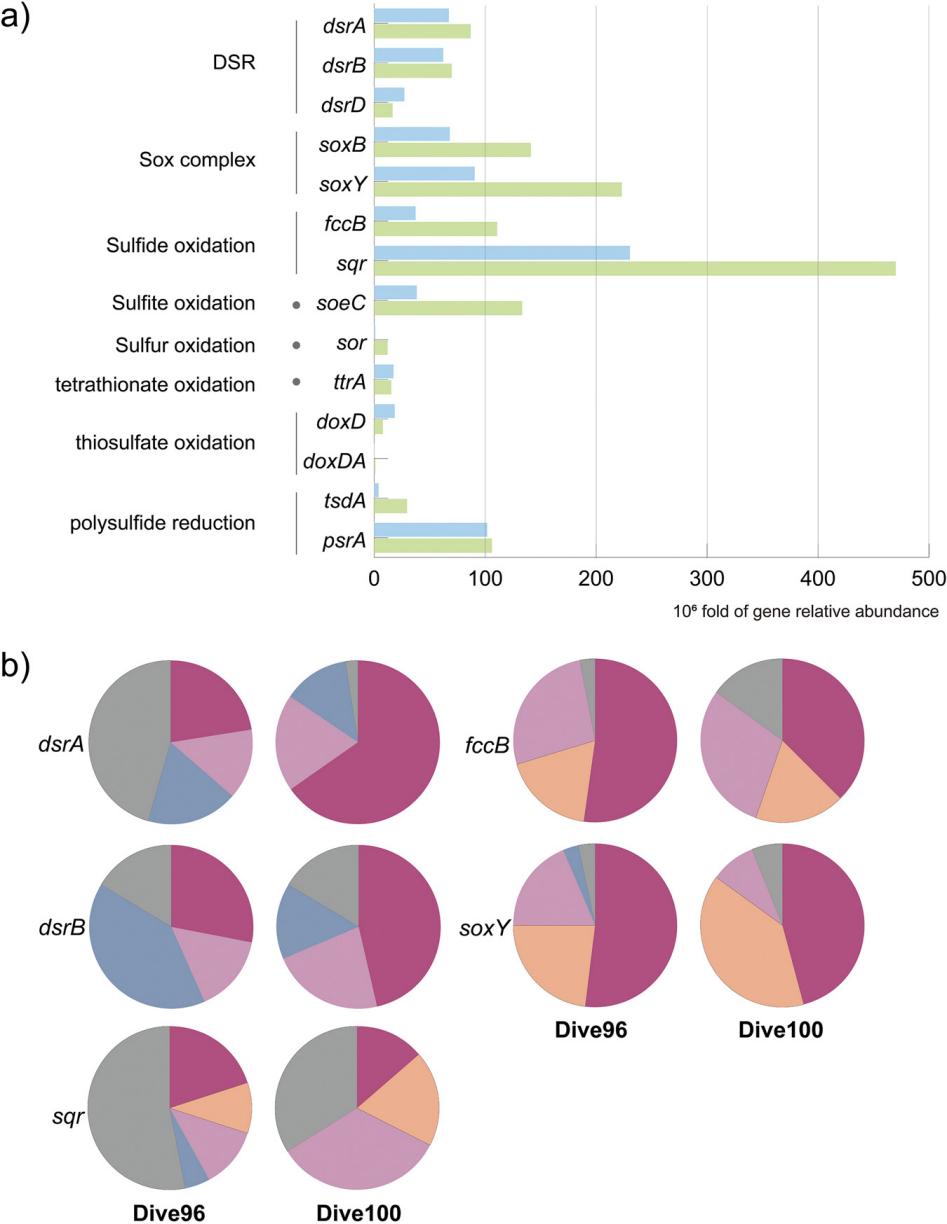
(a) Abundance statistics of sulfur metabolic genes in Dive96 and Dive100. (b) Taxonomic profile of key sulfur metabolic genes including *dsrAB*, *sqr*, *fccB*, s*oxY* in Dive96 and Dive100.

**FIG 3:**
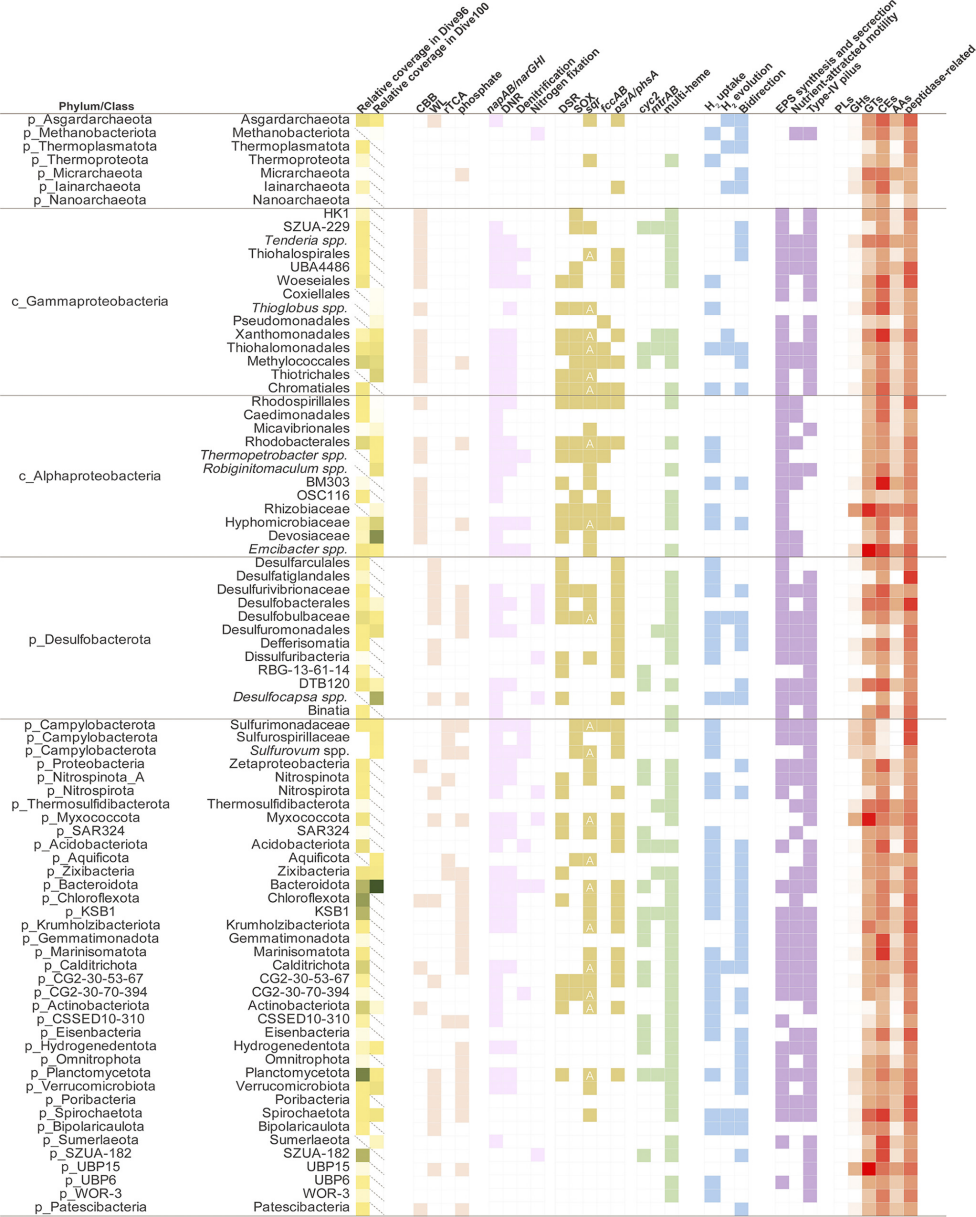
Statistics of abundance and metabolism potential of MAGs recovered in this study. In columns that presented the relative abundances of certain microbial groups in 2 samples, diagonal lines in grids indicated that certain microbial group was absent from MAGs pool assembled from the sample. “A” labeled in “sqr” column represented autotrophic subtypes (type IV, V, and VI) of sulfide: quinone oxidoreductase, while others were subtypes that were not indicated with capability of supporting autotrophic growth through sulfide oxidation.

Genes participating in sulfide and sulfur were enriched in both microbial communities, such as *sqr* (sulfide: quinone oxidoreductase) and *psrA* (thiosulfate reductase/polysulfide reductase chain A) (Fig. 2). Sulfide: quinone oxidoreductase was with the highest abundance in both samples among all genes involved in inorganic sulfur metabolism. It was also widely distributed in approximately half of MAGs associated to 2 archaeal phyla and 18 bacterial phyla. A total of 25.0% and 39.4% of the *sqr* abundance in Dive96 and Dive100, respectively, were contributed by subtypes (type IV, V, and VI) that play a role in microbial autotropic growth according to phylogenetic analysis (Fig. S2) (46). Sulfide dehydrogenase (*fccB*) was also with high relative abundance in Dive96 and consequently identified in MAGs associated with Alphaproteobacteria, Gammaproteobacteria, and Campylobacterota. In addition, *psrA* was enriched and encoded by 30.8% of MAGs in 16 bacterial phyla, indicating wide metabolic potential of thiosulfate or polysulfide reduction (Fig. 2). Extensive possession of *psrA* and *sqr* among different microorganisms also indicated the common metabolic potential for sulfur species (S^0^, S^2−^, S_2_O_3_^2−^) respiration or detoxification, which is beneficial for adaptation to the dynamic and toxic sulfide-rich environment. As for another key sulfur gene *dsrB* (dissimilatory sulfite reductase beta subunit), phylogenetic analysis revealed that oxidative type took up approximately 49.8% and 84.6% of total *dsrB* abundance in Dive96 and Dive100, respectively, emphasizing the dominance of sulfur oxidation (Fig. S3). In contrast, sequences of reductive *dsrB* took up less abundance and were mainly identified in MAGs associated to Desulfobacterota, Nitrospirota, and Zixibacteria (Fig. 3). Overall, genes associated with sulfur oxidation were relatively enriched in comparison to sulfate reduction ones (Fig. 2), suggesting that the microbial sulfur oxidation might be prior to sulfate reduction.

Taxonomic assignment of key sulfur genes *sqr*, *fccB*, *dsrAB* and *soxY* revealed the major players of microbial sulfur cycle in Dive96 and Dive100 (Fig. 2). Alphaproteobacteria, Campylobacterota, Gammaproteobacteria, and Desulfobacterota were the dominant contributors for sulfur-related genes. Total abundance of sulfur genes assigned to these microbial groups ranged from 46.6% to 97.6%, suggesting they were the dominant sulfur metabolic groups in Dive96 and Dive100. Contribution of Gammaproteobacteria to all 5 genes was the highest in both communities, with abundance taking up 33.9% and 41.9% on average. Following microbial groups were Campylobacterota, with average contribution to *fccB*, *soxY* and *sqr* reaching 16.8% and 25.8% in Dive96 and Dive100, respectively, while no DsrAB sequence were assigned to Campylobacterota (Fig. 2 and Fig. 3). Alphaproteobacteria was also an important contributor, averaging 16.8% (Dive96) and 22.9% (Dive100) of 5 sulfur genes (*sqr*, *fccB*, *dsrAB,* and *soxY*) that were taxonomically assigned to it. In addition, 26.9% (Dive96) and 14.1% (Dive100) of DsrAB protein sequences were assumed to be taxonomically related to Desulfobacterota, corresponding to the reductive type of DsrB sequences identified phylogenetic analysis (Fig. 3 and Fig. S3). A few reductive types of DsrB sequences was also identified in Nitrospirota, Chlofoflexota, Planctomycetota, and Zixibacteria associated MAGs. Meanwhile, Desulfobacterota also contributed about 3.2% of *soxY*, suggesting it might comprise sulfur-oxidizing members (Fig. 2 and Fig. 3).

Diverse MAGs, encoding *dsrABEFHLMKJOP* gene clusters or Sox thiosulfate oxidation pathway, were also recovered in Dive96 and Dive100, including Gammaproteobacteria (*Thioglobus*, UBA4486, SZUA-229 Thiohalomonadales, Thiotrichaceae, Woeseiales, and Xanthomonadales) and Alphaproteobacteria (Rhodobacteraceae, Rhodospirillales, and Rhizobiales) (Fig. 3). Co-occurrence of the reverse dissimilatory sulfate reduction pathway (rDSR) and Sox pathway indicated the metabolic potential for sulfide and thiosulfate oxidation by various Alphaproteobacteria and Gammaproteobacteria members. Sox gene clusters were also identified in Camoylobacterota (*Sulfurovum*, Sulfurospirillaceae, Sulfurimonadaceae), Aquificota, and Desulfobacterota. The presence of the Sox pathway in Desulfobacterota-associated members, such as Desulfobulbaceae and Desulfurivibrionaceae, has been reported in sulfide mineral community (47), which might represent novel cable bacteria species that were able to form large filaments for sulfide oxidation and thrive across anoxic and oxic niches (48) (Fig. 3). *soxCD* was absent from some of the putative sulfur-oxidizing members, including Desulfobacterota, Hyphomicrobiaceae, *Thiolapillus*, SZUA-229, and Xanthomonadales, indicating incomplete oxidation of thiosulfate and production of elemental sulfur. However, Hyphomicrobiaceae might be capable of disproportion of elemental sulfur as *sor* (sulfur oxygenase) was also identified (Fig. 3).

### Microbial iron metabolic features.

**FIG 4:**
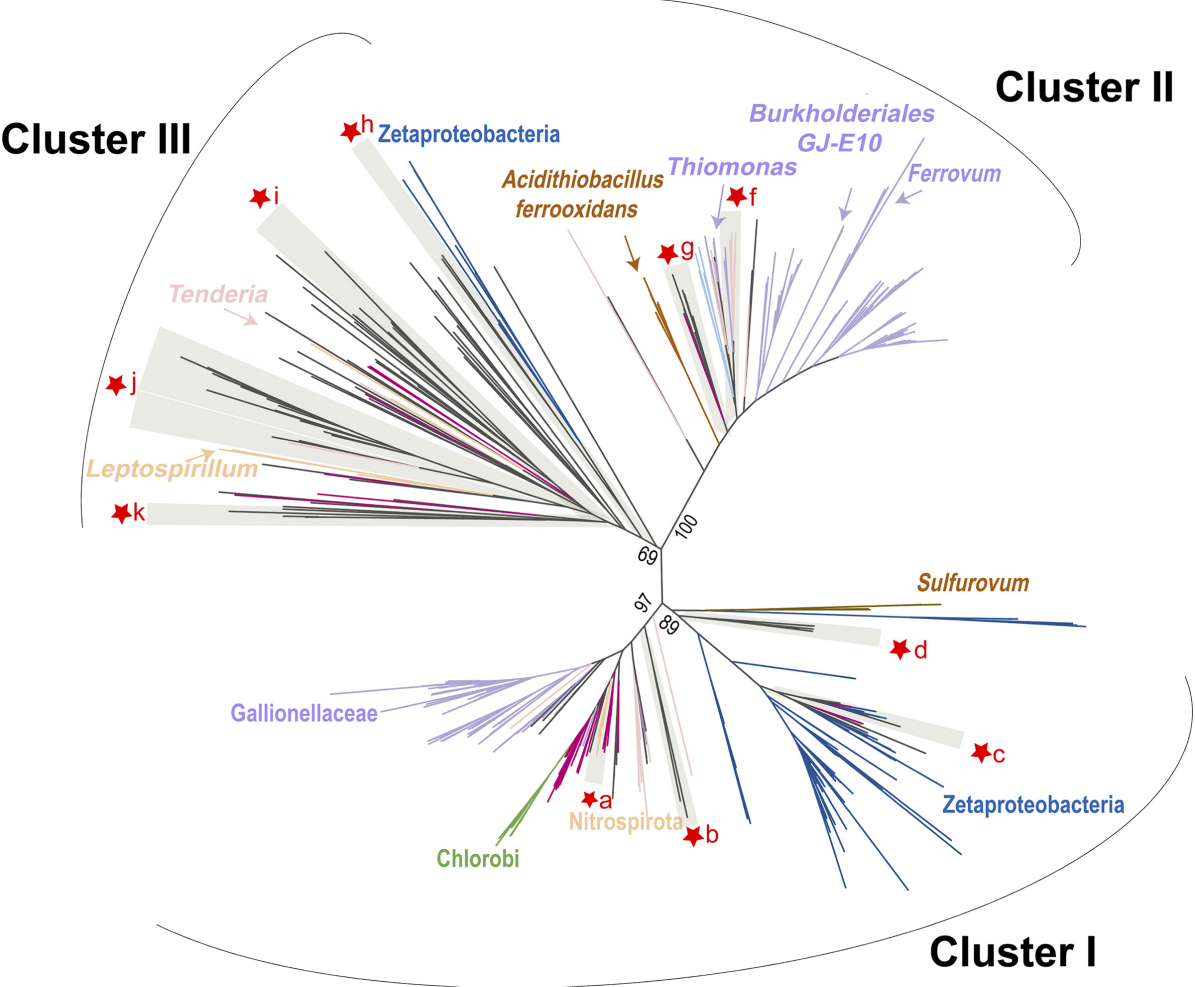
Phylogenetic tree of *Cyc2* and *Cyc2*-like protein sequences identified in this study. Visualization followed as McAllister et al., 2020 presented. Gray clusters that marked with star symbols indicated the positions novel *Cyc2* homologs identified in MAGs.

Here, homologs of the iron oxidase *Cytochrome c* Cyc2 were detected in 39 MAGs affiliated with 17 bacterial phyla, including Calditrichota, Eisenbacteria, Gemmatimonadota, Hydrogenedentota, Nitrospinota, Planctomycetota, Acidobacteriota, Krumholzibacteriota, CSSED10-310, KSB1, Gammaproteobacteria, Zetaproteobacteria, and Bacteroidota (Fig. 3, Table S2, and Fig. S9). Phylogenetic analysis revealed that 3 clusters were formed between these newly-discovered Cyc2 homologs and function-verified Cyc2 sequences, similar to the description in Mcallistar et al., (2020) (35) (Fig. 4). Represented by diverse FeOB including Zetaproteobacteria and *Leptospirillum* spp, cluster III comprised approximately 75% of Cyc2 homologs identified in this research. Most of the Cyc2-like sequences in cluster III were situated in a large subclade associated with acidophilic *Leptospirillum* spp and electroautotroph *Candidatus* Tenderia electrophaga, which has been reported to oxidize electrodes to derive energy (49). Four Cyc2 homologs from MAGs associated with DTB120 (Desulfobacterota) and Nitrospinota with situated in cluster I, which is represented by neutrophilic FeOB Gallionellaece. Cluster II also contained four Cyc2-like sequences identified in Bacteroidota and Methylomonadaceae (Gammaproteobacteria) associated MAGs.

Multi-heme proteins were widely detected among 64.6% of the recovered MAGs affiliated with 28 bacterial phyla, such as Gammaproteobacteria, Desulfobacterota, Calditrichota, and Gemmatimonadota (Fig. 3 and Table S2). These multi-heme sequences were inferred as cytochrome proteins by eggNOG 5.0 database. A total of 80.7% of these multi-heme protein sequences were predicted with signal peptide for secretion by SignalP (50), and 57.0% of them were presumed to be located on the outer membrane, periplasmic, or extracellularly by PSORTb (51), sites where multi-heme proteins have been found to conduct extracellular electron transfer during respiration activities (52).

Metabolic potential of iron acquisition and storage were massively detected among MAGs in Dive96 and Dive100 (Fig. S10 and Table S2). Approximately 22.4% of MAGs contained *amoA* (amonabactin biosynthetic gene) or *angR* (anguibactin system regulator), which are responsible for synthesizing amonabactin and anguibactin, siderophores with high chelation affinity to extracellular iron for metabolic usage. Transportation of siderophores was detected in over 60% of the MAGs, which is related to at least 7 different types of organic molecules such as anguibactin and bacillibactin. Fe(II) ions ABC transporter genes *FeoAB* were also detected in approximately 50% MAGs. Ferritin proteins serving in iron storage were densely identified in 68.2% and 59.8% of MAGs in Dive96 and Dive100, respectively (Fig. S10).

### Iron-oxidizing and iron-reducing microorganisms.

**(i) Zetaproteobacteria.** Identification of ZetaOTUs indicated the hidden, but large diversity of Zetaproteobacteria species inhabiting Dive96. A total of 21 types of ZetaOTUs were detected in Dive96, while 4 of them assumed as novel ZetaOTUs representing unassigned species (Fig. S5). Two types of ZetaOTU were detected in Dive100, related to members of *Mariprofundus* (ZetaOTU36) and *Ghiorsea* (ZetaOTU09)*. Ghiorsea* might be the dominant Zetaproteobacteria member in both Dive96 and Dive100, as ZetaOTU09 took up approximately 43.8% and 88.1% of the total Zetaproteobacteria abundance in Dive96 and Dive100, respectively. In addition, 5 MAGs associated with Zetaproteobacteria were also recovered from Dive96, and took up about 2.7% of the total abundance among all MAGs, indicating that energy derived from iron oxidation might also support chemosynthesis in vent chimneys (Fig. 3).

**(ii) Iron-reducing bacteria.** MAGs associated to iron-reducing representatives were recovered in Dive96 (Fig. 3), including Fe(III)-reducing and Mn(IV)-reducing *Deferrisoma* (44, 45) and ferrihydrite-reducing *Thermosulfidibacter* (42) (Fig. S6 and Fig. S7). Electrode-respiring and Fe(III)-reducing *Geopsychrobacter* (53) were detected in Dive96 and Dive100, which is also enriched in inactive sulfide mineral samples. In addition, genes *mtrAB* functioning in Fe(III) reduction (54) were also encoded by diverse MAGs associated to Acidobacteria, Desulfobacterota, Zixibacteria, etc. (Fig. 3 and Table S2). MAGs associated to Zixibacteria encoded both iron oxidoreductase *mtrAB* and rTCA pathways, indicating chemolithoautotrophic lifestyle. While previous research has indicated the iron-reducing metabolic potential for Zixibacteria in coastal sediment (55), the recovery of chemotrophic iron-reducing Zixibacteria in hydrothermal chemosynthetic ecosystem highlighted not only its wide distribution, but also function in iron and carbon biogeochemistry in global ecosystems.

**(iii) Magnetotactic bacterium.** Another MAG named Dive96_bin3.242, associated with Nitrospirota, also carried a complete magnetosome gene cluster and Wood-Ljungdahl pathway, which might be autotrophic magnetotactic bacterium (MTB) producing magnetosomes made of magnetite (Fe_3_O_4_) or greigite (Fe_3_S_4_) (Fig. 3 and Fig. S8). As a result of reductive sulfide-rich hydrothermal fluid and oxygenic seawater mixing, a dynamic redox gradient was created, and an oxic-anoxic interface was formed around the venting area, which might be an ideal niche for magnetotactic bacteria to grow and facilitate magnetosme production.

## DISCUSSION

### Mineral-involved chemosynthesis.

Sulfur oxidation was the most frequently detected pathway for chemosynthesis in Dive96 and Dive100. The majority of assimilatory RubisCO (Ribulose-bisphosphate carboxylase) sequences (form I and II [56]) were identified in MAGs carrying the rDSR or Sox pathway, such as Alphaproteobacteria and Gammaproteobacteria (Fig. 3). The reductive citrate cycle (rTCA) pathway was present in putative thiosulfate-oxidizing MAGs associated with Campylobacterota, Nitrospinota, and Aquificota in which a Sox cluster was also detected (Fig. 3). Overall, sulfur-oxidizing pathways were possessed by 64.10% of putative autotrophic MAGs in Dive96, and 88.46% in Dive100. Notably, some of these sulfur-oxidizing MAGs affiliated with Gammaproteobacteria and Alphaproteobacteria carried various combinations of sulfur-related genes such as *sqr*, Sox, rDSR, and *soeABC*, which indicated versatile genomic potential for sulfur oxidation using various sulfur substrates (sulfide, thiosulfate, sulfite) in multiple reaction sites (cytoplasmic and periplasmic), connecting energy conservation with cellular growth and primary production (57). These sulfur-oxidizing bacteria (SOB) such as Thiohalomonadales, Thiotrichaceae, and Hyphomicrobiaceae were enriched in microbial communities, further emphasizing the significance of sulfur oxidation in microbial chemosynthesis (Fig. 3).

Some of the sulfur-oxidizing primary producers were also detected in microbial communities on inactive sulfide minerals (Fig. S4). After extinction of hydrothermal vent activities, solidate sulfide mineral was presumed to substantially support chemosynthesis in terms of microbial sulfur-oxidation. Autotrophic sulfur-oxidizing bacteria enriched in inactive sulfide minerals might be experts in utilizing sulfide mineral as energy source, capable of dissolving or extracting reductive sulfide species from sulfide minerals for energy conservation. Members of Thiohalomonadales encoding Sox and rDSR gene clusters were detected in Dive96 and Dive100, which were also previously found enriched in extinct vents in the East Pacific Rise and sulfide mineral in Manus Basin (58, 59). Sulfur-oxidizing SZUA-229 members dominant in inactive sulfide minerals or chimney walls (58, 59) were also recovered in Dive96, with MAGs encoding Sox gene clusters. Electrode-respiring *Cadidatus Tenderia* was recovered in Dive96 although the sulfur-oxidizing pathway was absent. Detection of these “mineral-preferred” sulfur-oxidizing Gammaproteobacteria indicated chimney mineral supporting microbial chemosynthesis in Dive96 and Dive100 (Table 1, Fig. 3, and Fig. S4). With the additional assistance of diverse “mineral” Gammaproteobacteria members, microbial community might be able to utilize sulfur from chimney minerals and surrounding fluid for chemosynthesis, simultaneously.

**TABLE 1:** Feature and distribution of several Gammaproteobacteria members found in Dive96 and Dive100^*a*^

Class/phylum	Members	Sample	Reference
Gammaproteobacteria	SZUA-229	Dive96	47, 58, 59
Gammaproteobacteria	*Tenderia*	Dive96	58, 59
Gammaproteobacteria	Thiohalomonadales	Dive96, Dive100	58, 59
Gammaproteobacteria	Xanthomonadales	Dive96, Dive100	48, 58

a “Mineral” type Gammaproteobacteria are presumed capable of oxidizing insoluble sulfur from chimney minerals except for *Tenderia*. Phylogenetic positions with reference Gammaproteobacteria genomes from other hydrothermal vents or inactive minerals were presented in Fig. S4.

### Expanded mineral-microbe interaction.

Microbial sulfur and iron metabolisms could be significant in as both chimney samples were composed of sulfide minerals (Table S1 and Fig. S1). As both *fccB* and *sqr* participate in the oxidation of sulfide and generation of elemental sulfur product (S_8_ or HS-(S_n_)-SH) in forms of sulfur globules or extracellular organic minerals (53, 54), their significant enrichment also highlighted the metabolic potential in oxidizing sulfide and transforming sulfur-related minerals in the microbial community. Through sulfur metabolisms, microbes might participate in sulfide material transformation involved with mineral weathering as a result of energy conservation or cellular detoxification.

Electron transportation between cells and minerals was also fascinating as it indicated microorganisms’ capabilities of utilizing mineral for energy conservation. Previous metagenomic surveys have demonstrated that putative extracellular electron transporter such as multi-heme proteins (36, 52, 56) were frequently detected and highly expressed in microbial communities accommodated on inactive sulfide minerals (58). In Dive96 and Dive100, wide distributions of multi-heme protein sequences were also observed, and their products were mostly assumed to be secreted to periplasmic or extracellular sites where oxidoreduction were reported (52). This expanded distribution of multi-heme proteins (Fig. 3 and Table S2) covered both chemolithoautorophs and heterotrophs with different energy conservation strategies such as SOB (Gammaproteobacteria, Campylobacterota), sulfate-reducing bacteria (SRB, Desulfobacterota), nitrate-oxidizing bacteria (NOB, Nitrospinota), and iron-oxidizing bacteria (FeOB, Zetaproteobacteria and Zixibacteria). As potentially serving in establishments of massive networks of extracellular electron transfer (EET) between cells or cells to minerals (36, 52, 56), multi-heme proteins can assist in cooperative energy conservation between syntrophic partners (57) or enabling microorganisms to access more diverse electron acceptors or donors, such as insoluble sulfide minerals or soluble Fe(III), Mn(IV) ions, etc. (36, 52, 56). The establishment of well-organized electron transport networks by multi-heme proteins might comprehensively enhance microorganisms’ adaptation and survival in extreme hydrothermal conditions (37), extending their energy sources to solid chimney minerals rather than being constrained by the availability of materials from hydrothermal fluids or seawater.

### Potential influences of minerals on microbial community.

**FIG 5:**
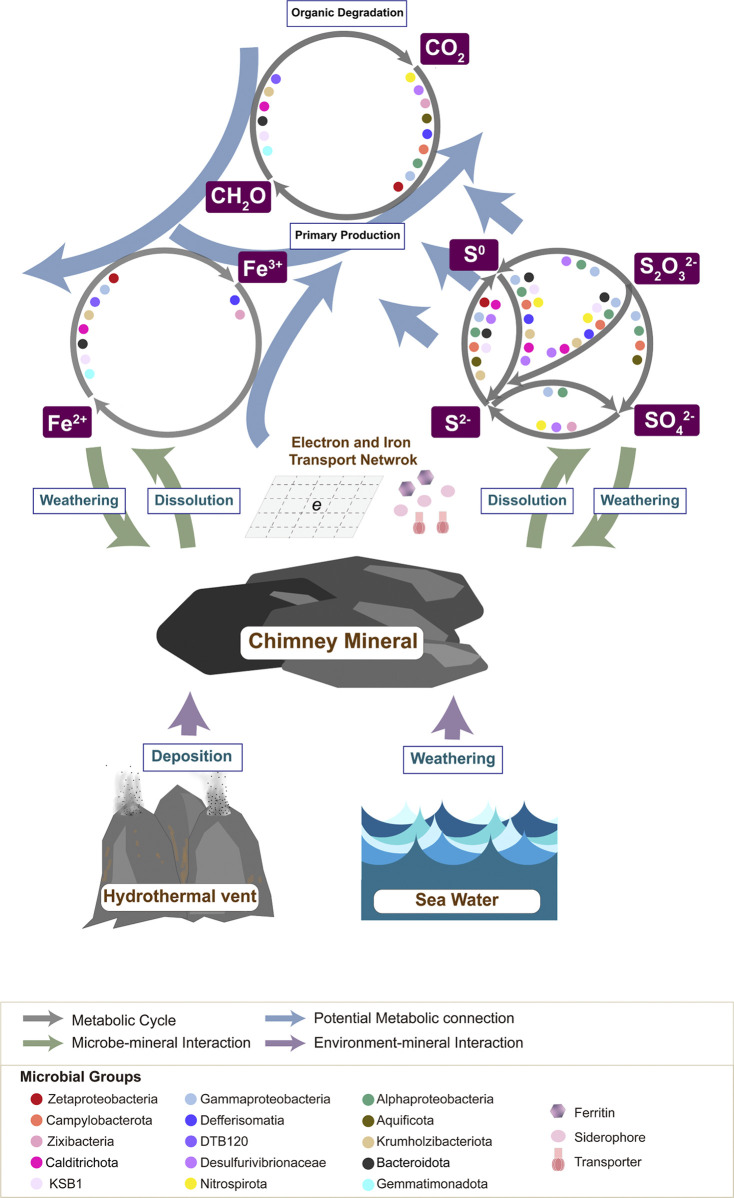
Potential scheme of microbial sulfur and iron metabolisms and associated interactions between hydrothermal environment, chimney mineral and microbial communities in Longqi region.

Distinct microbial iron oxidoreduction metabolic potential was observed between Dive96 and Dive100, and more diverse microbial participation was featured in Dive96. Firstly, a more diverse composition of iron-oxidizing Zetaproteobacteria represented by ZetaOTUs was detected in Dive96 (Fig. S5). Secondly, more diverse Cyc2 homologs possessed by MAGs were also detected in Dive96 than in Dive100 (Fig. 4 and Fig. S9). Thirdly, various iron-related bacteria were also identified in Dive96, such as Defferisomatia, Thermosulfidibacter, Desulfovibrionaceae, and magnetotactic bacteria Nitrospirota (Fig. 3, Fig. 5, Fig. S6, Fig. S7, and Fig. S8) (42, 44, 45, 60, 61). This increased diversity of both iron-oxidizing members and iron-reducing members might indicate a more active iron biogeochemical cycle in Dive96 directed by multiple microbial species.

One of the underlying reasons could be owed to the difference in mineral compositions between Dive96 and Dive100. Participation of chimney minerals in chemosynthetic sulfur oxidation was already indicated by the detection of abundant mineral-based Gammproteobacteria members in Dive96 and Dive100. In fact, mineral composition could affect local microbial structure (62–68) and cause thermodynamic shifts in the local environment (69). Previous research has illustrated the significant influence of pyrrhotite on microbial iron-oxidizing metabolism in mineral-based communities (70). Content of pyrrhotite supplied to the sphalerite mixture provided more dissolved iron to surrounding environments, which subsequently allowed iron-oxidizing members to thrive and enhance biotic iron oxidization and sulfur oxidization in the microbial community (70) (Fig. 5). In Dive96, it is possible that the augment of Fe(II) and Fe(III) supplied from pyrrhotite enhanced the growth of more diverse FeOBs, such as Zetaproteobacteria. Further dissolution of minerals also released sulfur materials, which could enhance in chemosynthetic sulfur oxidation in Dive96 and allow potential heterotrophic FeOBs to thrive. Accelerated FeOBs further caused dissolution and oxidation of minerals, forming positive feedbacks with increased Fe(III) supply and growth of iron-reducing bacteria. As a result, more diverse microbial iron oxidoreduction indicated by the composition of FeOBs and iron-reducing species was observed in Dive96.

### Novel iron-oxidizing bacteria.

As Cyc2 functions as an iron oxidase in FeOBs (34), its distribution in microorganisms might be indicative of potential iron-related functions in hydrothermal vent microorganisms. However, as functional understandings about Cyc2 proteins are still very limited, caution should be practiced about connecting the possessions of Cyc2 homologs and biotic iron oxidation among microorganisms. In this study, multiple steps of bioinformatic analysis were conducted to demonstrate the proper annotation of Cyc2 homologs. Firstly, close examination of conserved regions with heme-binding functions (71) were required. Only sequences with N terminus composition similar to those encoded by function-verified iron-oxidizing bacteria, such as *Gallionella*, *Mariprofundus*, *Thiomonas,* and *Acidithiobacillus* spp. (Fig. S9) could be identified as Cyc2 homolog candidates (33, 35, 72, 73). Secondly, checking the destinations of these putative Cyc2 products was also necessary, as current research supported that Cyc2 conducted Fe(II) oxidation on the outer membrane where energy conservation was performed with cytochrome oxidases in FeOBs (33, 74, 75).

Analysis in the conserved region compositions, product destinations, and phylogeny diversity revealed that features of novel Cyc2 homologs catered to the current understandings of Cyc2 proteins verified for iron oxidation. Given their similarities, it is theoretically possible that these protein products function as an iron oxidases or electron transporter as previously demonstrated (34, 76), further enhancing microorganisms′ capabilities of conducting extracellular electron uptake or even allowing them to access additional electron donors such as Fe(II) for energy conservation (77).

In this way, potential FeOBs encoding Cyc2 were identified in Dive96 and Dive100. Some of the relevant MAGs also encoded carbon fixation pathways and might be potential chemolithoautotrophic iron-oxidizing bacteria. In addition to Zetaproteobacteria discussed above, 2 MAGs related to Gammaproteobacteria (class SZUA-229 and Thiohalomondales) carried both Cyc2 homologs from cluster III and assimilatory form of RubisCO sequences (Fig. 3 and Fig. 4). These autotrophic MAGs also carried metabolic potential for nitrate reduction and thus catered to the definition of NRFeOx (29), which is capable of utilizing nitrate as electron acceptor coupling with iron oxidation. Our result expands the diversity of uncultured iron-oxidizing Gammaproteobacteria inhabiting in hydrothermal vent regions, in addition to isolated *Thiomicrospira* (26).

Notably, the majority of novel Cyc2-like sequences were found in putative heterotrophic bacterial groups including DTB120, Calditrichota, Gemmatimonadota, Eisenbacteria, Hydrogenedentota, Krumholzibacteriota, Planctomycetota, CSSED10-310, Acidobacteriota, Bacteroidota, KSB1, and SAR324.

Apart from Bacteroidota (78, 79) and uncultured phylum DTB120 (28), other heterotrophic groups detected with Cyc2 here have not been presumed with iron oxidation function previously. However, functional profiles of these MAGs in this study were identical to the prediction of uncultured iron-oxidizing extremophiles featured with functions of versatile organic degradation and utilization of nitrate as electron acceptors (31). For one thing, these MAGs encoded enriched copies of carbohydrate-active enzymes (CAZymes) while absent of carbon fixation pathways. For example, MAGs related to Gemmatimonadota contained up to 20 copies of PLs (polysaccharide lyases), while members of CSSED10-310, Krumholzibacteriota, Planctomycetota, Hydrogenedentota, Calditrichota, and Bacteroidota contained approximately 30 copies of CEs (carbohydrate esterases), on average. MAGs associated with Eisenbacteria also carried over 50 copies of genes functioning in peptide degradation, suggesting its specialty in utilizing peptide molecules (Fig. 3). Furthermore, genes involved in nitrate or nitrite reduction were also carried by these MAGs, suggesting that nitrate may serve as electron acceptors for microbial iron oxidation. Metabolisms and functions that were practical for microbial iron oxidation were also encoded among these microbial groups. Sixteen MAGs associated with Krumholzibacteriota, Acidobacteriota, Bacteroidota, Calditrichota, CSSED10-310, Hydrogenedentota, Eisenbacteria, Planctomycetota, KSB1, and SAR324 also encoded *cytochrome c* oxidase for respiration (Table S2), indicating the complete energy conservation path known for microbial iron oxidation (35) (Fig. 3). Functions that were potentially advantageous for iron oxidation were also found, including EPS production and biofilm generation, which can protect microorganisms from suicidal cell encrustation caused by iron-oxyhydroxide (80, 81), and enhance their efficiency in acquisition of organic carbon and iron as aggregates (82).

In fact, microorganisms with phenotypes of heterotrophic iron oxidation have been reported in species of *Marinobacter* (83–86), *Alteromonas*, *Pseudoalteromonas*, Pseudomonas, *Halomonas*, and *Alcanivorax* (87–89), which were indicated with important roles in the iron cycle in subsurface fluid and hydrothermal vents. Research targeting microbial mats covering hydrothermal chimneys have also suggested the activity of heterotrophic iron oxidizers DTB120, and play an important role in element cycling (28). Similarly, our study also indicated the potential function of iron oxidation in diverse heterotrophic bacteria. From the perspective of hydrothermal vent biogeochemistry, a novel biogeochemical pathway connecting biotic dissolution or weathering of sulfide minerals and accessibility of organic carbon was predicted because of the presence of heterotrophic FeOBs. While taking advantage from iron oxidation, heterotrophic FeOBs also cause further dissolution of sulfide minerals as the equilibrium of mineral dissolution is constantly disrupted. Additional sulfur species such as polysulfide, sulfide, and thiosulfate could be supplied into microbial communities (90, 91), and further accelerate carbon fixation performed by sulfur-oxidizing bacteria such as Gammaproteobacteria and Campylobacteria (26). In this way, iron-oxidizing heterotrophs and sulfur-oxidizing autotrophs are mutualistic to each other as they participate in the material replenishment processes for counterparts as a result of cellular energy conservation. A new iron-related biogeochemical pathway influenced by organic carbon availability may be present, which is under the control of the interaction of sulfide-oxidizing chemolithoautotrophs and iron-oxidizing chemoorganotrophs.

### Conclusion.

In this study, a metagenomic survey was conducted targeting 2 Longqi hydrothermal vents in the SWIR. Through bulk metagenomic analysis and functional profiling on 295 MAGs, microbial structure and metabolic features in Longqi hydrothermal vents revealed the potential impacts of fluid-water mixing and mineralogical composition on microbial communities. Sulfur oxidation by diverse Gammaproteobacteria, Alphaproteobacteria, and Campylobacterota might be the major energy source for primary production in both active black smoker and diffuse vent chimneys, while support from chimney mineral for microbial chemosynthesis was also emphasized. Novel chemoheterotrophic iron-oxidizing species in 12 phyla were identified, and might use nitrate as electron acceptors to couple with iron. Wide distribution of multi-heme and siderophore transportation genes among MAGs also suggested massive electron transportation network between microbes and chimney minerals. Stronger microbial iron oxidoreduction potential was revealed in Dive96 as more diverse compositions of iron-oxidizing and iron-reducing bacteria were detected. It is possible that pyrrhotite in Dive96 minerals provides more dissoluble iron supply for the growth of iron-related species. Inhabitance of these novel iron-oxidizing chemoheterotrophs could further influence iron biogeochemistry in hydrothermal vent minerals but more efforts are essential to verify and quantitively study their iron-oxidizing capabilities.

## MATERIALS AND METHODS

### Sample collection and mineralogical analysis.

**FIG 6:**
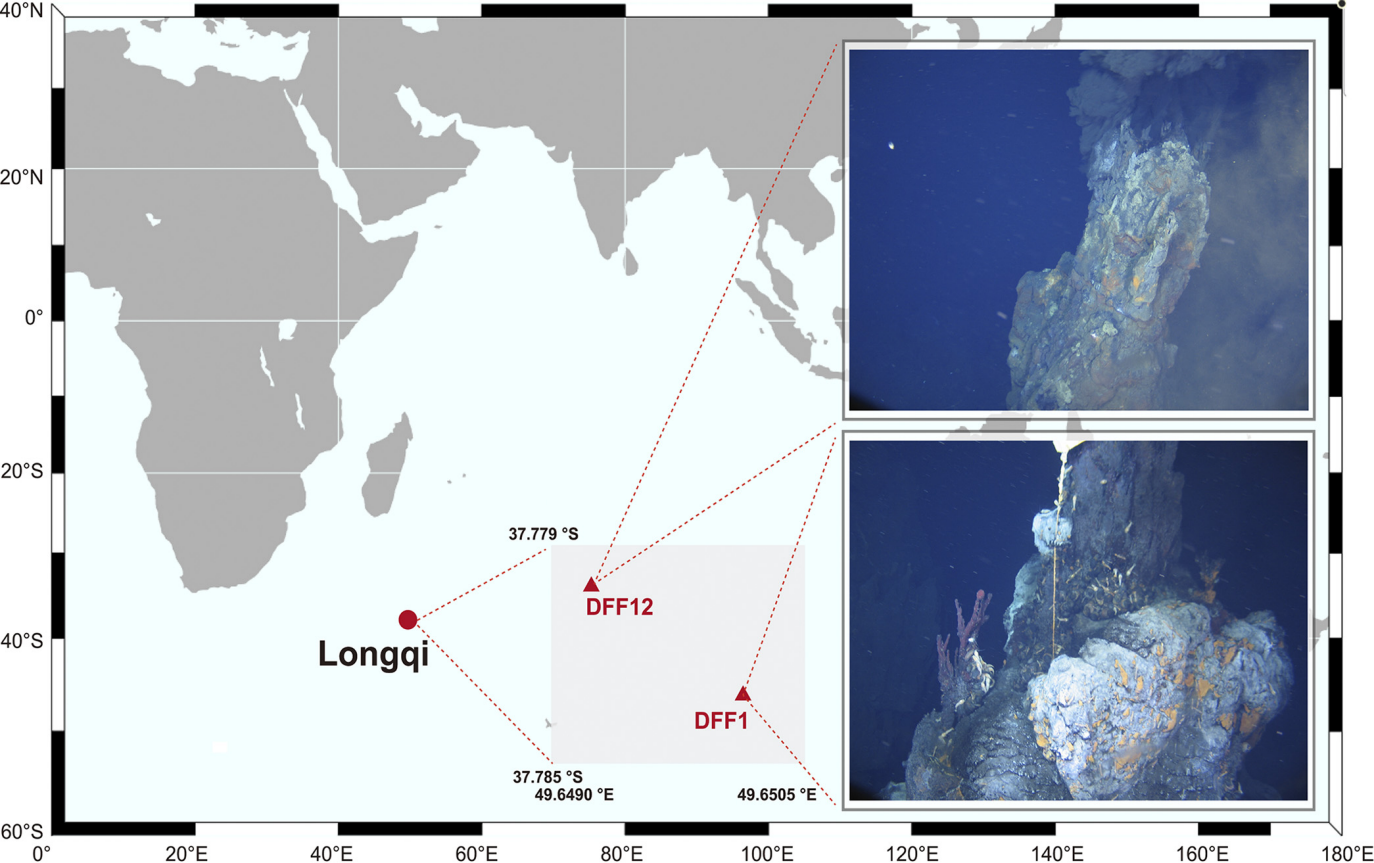
Geographical location of Longqi hydrothermal vent region and images of hydrothermal vents DFF12 and DFF1.

Samples were collected in 2015 on the Chinese Dayang 35^th^ cruise by the R/V, *Xiangyanghong 9,* and Human operated vehicle (HOV), *Jiaolong,* using a seven-function manipulator and sample basket. Sample Dive96 was collected from the exterior of the vent DFF12 in Longqi (Fig. 6). Sample Dive100 was collected from a spire branch from the vent DFF1 (also named Jabberwocky). When aboard, the 2 samples were washed using sterile seawater and stored at −80°C until lab subsampling for metagenomic sequencing and mineralogical analysis took place.

Bulk sulfide mineral Dive96 seemed homogeneous with constant mineral composition when observed with naked eyes. Thus, it was cut into 2 small sections for mineralogical analysis and metagenomic sequencing, respectively. Dive100 was a hollow branch and accidentally broken apart during transportation. Only intact pieces of this branch mineral were chosen for further metagenomic sequencing and mineralogical analysis. To conduct mineralogical analysis, a fraction of mineral sample was cut apart and dried out for grating. Three grams of mineral powders with particle sizes less than 0.074 mm were then examined by Powder X-ray Diffraction (X’ Pert PRO) under diffraction conditions of copper target, 45 KV tube voltage, 40 mA tube current, scanning step size at 0.0167°/2θ, scanning range of 5–80°/2θ, and scanning speed at 1.8°min^−1^. Mineralogical composition was analyzed using the Rietveld full spectrum fitting method (92).

### Metagenomic sequencing, assembly, and binning.

Magnetic Soil and Stool DNA Kit (TIANGEN) was used for DNA extraction for samples Dive100 and Dive96. For sample Dive96, a second extraction was conducted following modified DNA extraction protocols introduced by Jenni et al. (93), and products from 2 extraction procedures were mixed to obtain enough DNA for metagenomic sequencing. Library construction was performed using NEBNext Ultra DNA Library Prep Kit for illumina. Sequencing was conducted on the Illumina Hiseq X-10 (PE150) platform in Novogene Bioinformatics Technology Co., Ltd., Beijing, China.

Raw sequencing data were filtered by Trimmomatic (94) and assembled through IDBA-UD with parameters as “–mink 50,–maxk 92,–steps 8”. MetaBAT2 with 12 sets of parameters was used for metagenomic binning, and standard pipeline of DASTools was used to recover non-redundant binning genomes (95, 96). Subsequent MAG quality checks and refinements were conducted by checkM and refineM (97, 98), respectively. Only bins with genome completeness higher than 70% and contamination less than 10% were retained for further analysis.

### Phylogeny and abundance profile of MAGs.

Genome classifier GTDB-Tk (database version: Release 95) (99) was used to assign taxonomy of MAGs. For phylogeny analysis, additional 201 GTDB representative genomes were downloaded and included in phylogenetic analysis (100). Sixteen ribosomal proteins (L2, L3, L4, L5, L6, L14, L16, L18, L22, L24, S3, S8, S10, S17, and S19) were identified by PhyloSift (101) and aligned by MUSCLE (102). Concatenated alignment of 16 ribosomal proteins were merged (103), where positions that contained more than 30% gaps were removed by trimAI (104). Reduced alignments were used as input for IQ-TREE (105, 106) and phylogenetic trees were constructed with a bootstrap value set at 1000 and amino acid substitution model set at LG+G, as Hug et al. suggested (103). Abundance of MAGs was calculated by the proportion of coverage of their binned contigs in the sum of coverage of all contigs assembled in the metagenome. BWA-MEM were used in metagenomic reads mapping to MAGs, and Samtools was used to sort the mapping results (107, 108), which were ultimately analyzed by bedtools to obtain the relative coverage of each MAG (109).

### Basic gene calling and multi-step functional annotation.

Multiple annotation tools were used in this study. Coding sequences were first called by Prokka (110). First-round annotation was based on KEGG (111, 112) and eggNOG 5.0 (113, 114). ko2cog tool (http://www.genome.jp/kegg/files/ko2cog.xl) was used to assign COG predictions generated from eggNOG annotation into KO numbers. In the second round, the Hidden Markov Search (115) was conducted as a complementary and double-check measure. Alignments or pre-built models were obtained from TIGRFAMs (116), Pfam (117), CDD (118), and COG (119). These searches were limited at 1 × 10^−20^ as cutoff *e* value. To identify hydrogen metabolism genes, custom Hidden Markov Models for hydrogenases from Lithogenie (https://github.com/Arkadiy-Garber/LithoGenie) were deployed, following recommended bitscore cutoffs. Further affirmation in HydDB (120) was also performed for these candidate sequences. CAZymes were annotated using dbcan2 package with default settings (121). Genes related to protein degradation were searched, as Baker et al. suggested (55), in which transcriptional regulator (PF0155) was neglected.

Homologs of iron oxidase Cyc2 were first identified by Lithogenie. Examinations were performed based on alignment features at N-terminal region and predictions of subcellular localizations. Subcellular locations of their products were predicted using SignalP 5.0 (50) and PSORTb 3.0 (51). Only those sequences with similar features that He et al., reported (122) and potentially localized in periplasmic would be recognized as Cyc2-like sequences for further discussion. Multi-heme proteins were predicted as Meier et al. suggested (58), with doubled CXXCH motif PF09699. Genes related to siderophore synthesis and iron transport were annotated using Fegenie (38) with recommended thresholds. Magnetosome associated genes were annotated using the NCBI RefSeq non-redundant protein database (downloaded in September, 2020) (123).

### Phylogenetic analysis and abundance profile of functional genes.

Classification of Rps3 protein sequences were based on the NCBI RefSeq non-redundant protein database (downloaded in September, 2020) (123). Maximum likelihood trees of SQR, DsrB, Cyc2, and hydrogenase protein sequences were constructed using IQ-TREE (105, 106). Due to the large numbers of annotated sequences, SQR and DsrB amino acid sequences were first clustered using cd-hit (124) with identity threshold set at 0.85 and 0.75, respectively. Representatives were then merged with reference sequences (125–127) and aligned using Muscle (102). Positions with more than 30% gaps would be discarded by trimAI (104). Amino acid substitution models for SQR and DsrB alignment results were inferred through ModelFinder (46). As for Cyc2 phylogeny, additional homologs were searched as Mcallistar et al. (35) suggested, which yielded 484 sequences from IMG and NCBI databases. IQ-TREE ultrafast bootstrapping algorithm (105, 106) and VT+F+R9 models were utilized to construct the phylogenetic tree of these homologs and Cyc2-like sequences found in this study. Brief annotations of resulted phylogenetic trees were conducted in the online tool, iTOL2 (128).

For Rps3 protein sequences, abundance was determined by the relative abundance of the situating contig. For other functional genes, abundance was calculated based on their proportion of recruited reads in the proportion of total reads, as Hou et al. suggested (59). BWA-MEM and Samtools were used in reads mapping and sorting processes (107, 108) while bedtools (109) was used for relative coverage calculation.

### ZetaOTU identification and classification.

ZetaOTUs in unbinned contigs were considered as a way of investigating the composition of Zetaproteobacteria, given the difficulty of simultaneously recovering Zetaproteobacteria genomes and corresponding 16S rRNA sequences (107). All 16S rRNA sequences in assembled contigs were first identified by metaxa2 (129). Primary classification was completed through blastn with the SILVA database (version: 138.1) (130, 131). Sequences assumed to be Zetaproteobacteria-related were then classified using ZetaHunter (39).

### Data availability.

All metagenome-assembled genomes are available under NCBI Bioproject PRJNA771178.
