# Supplementary material for: Metagenomic Features Characterized with Microbial Iron Oxidoreduction and Mineral Interaction in Southwest Indian Ridge
Source: Microbiol Spectr. 2022 Oct 26;10(6):e00614-22. doi: 10.1128/spectrum.00614-22 (PMC9769843; doi:10.1128/spectrum.00614-22)
Supplement: Supplemental file 2 — Supplemental material. Download spectrum.00614-22-s0002.pdf, PDF file, 1.3 MB [file spectrum.00614-22-s0002.pdf]

Supplementary Table 1. Geological information and metagenomic sequencing results of Dive96 and Dive100.

Information	Characteristic	Sample	
		Dive96	Dive100
Geological metadata	Vent	DFF12	DFF1
	Status of Vent	Active black smoker	Diffuse vent
	Time of sampling	2015.01	2015.02
	Depth	2759 m	2796 m
	Longitude	49.648642 °E	49.649967 °E
	Latitude	37.780157 °S	37.783283 °S
	Mineralogical composition	Sphalerite, gypsum, pyrrhotite	Sphalerite
Metagenomic Sequencing	Clean data	149.8 G	141.2 G
	Contigs	4.6 G	1.1 G
	Number of all MAGs	660	212
	Number of mid-quality MAGs	208	87

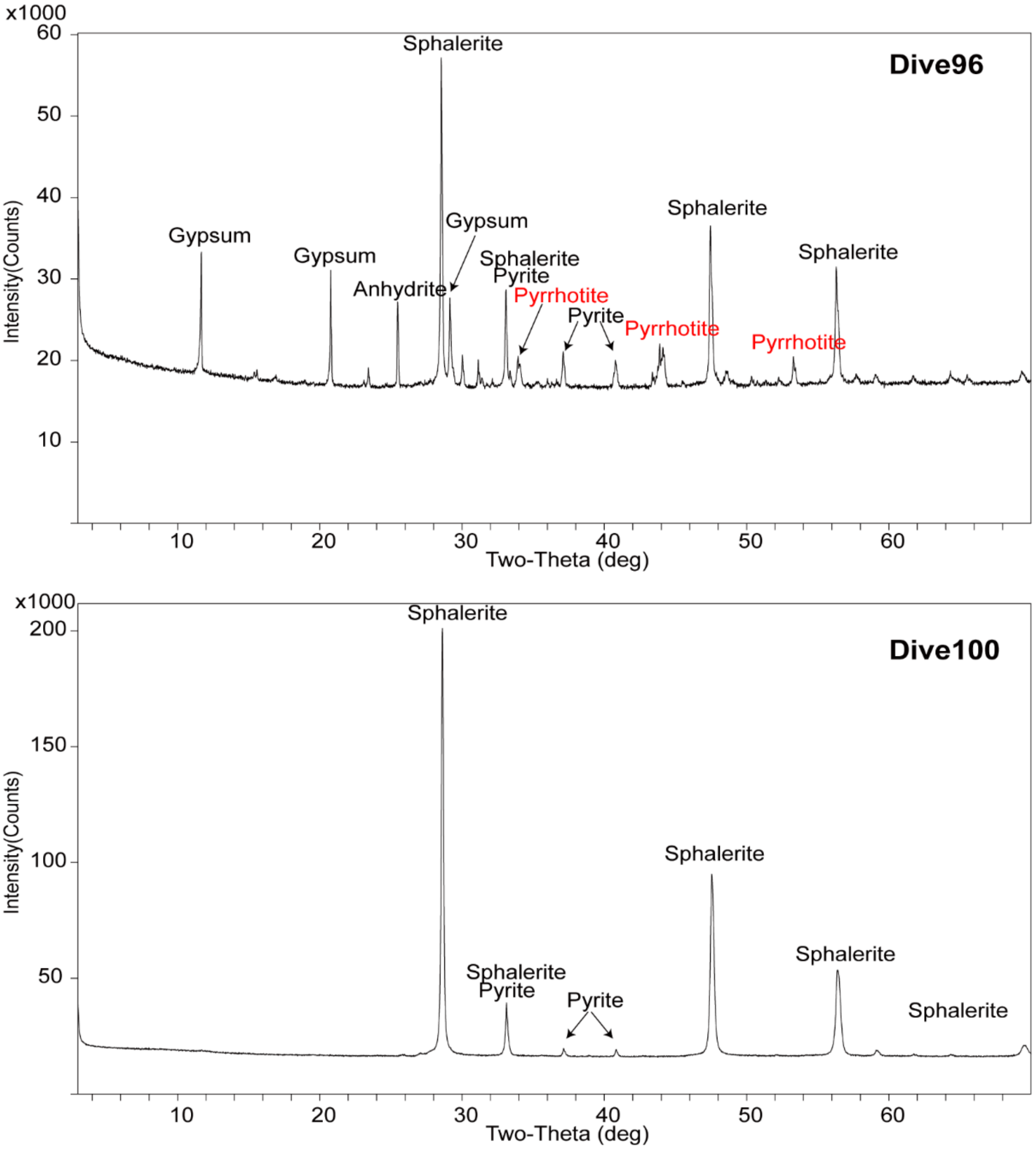

Supplementary Figure 1. XRD results of Dive96 and Dive100.

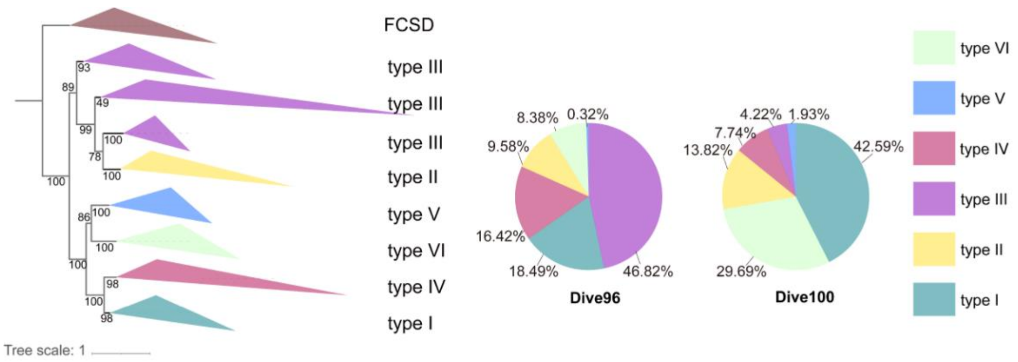

Supplementary Figure 2. Phylogenetic tree and abundance profile of SQR protein sequences identified in this study.

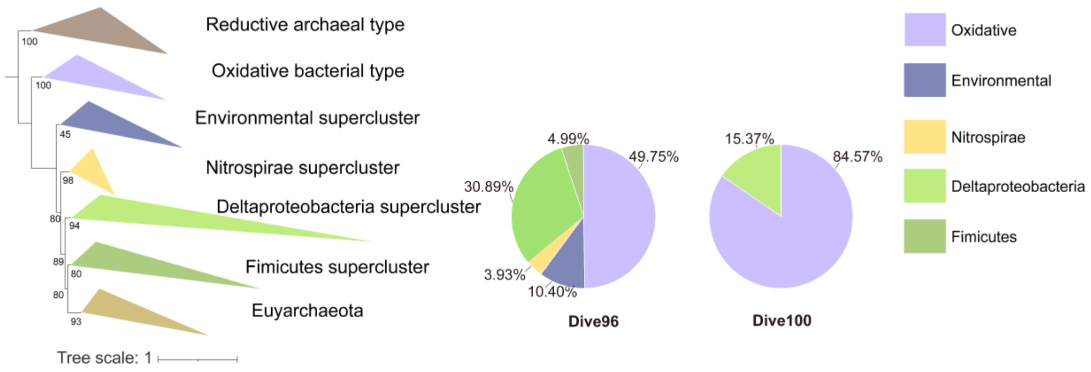

Supplementary Figure 3. Phylogenetic tree and abundance profile of DsrB protein sequences identified in this study.

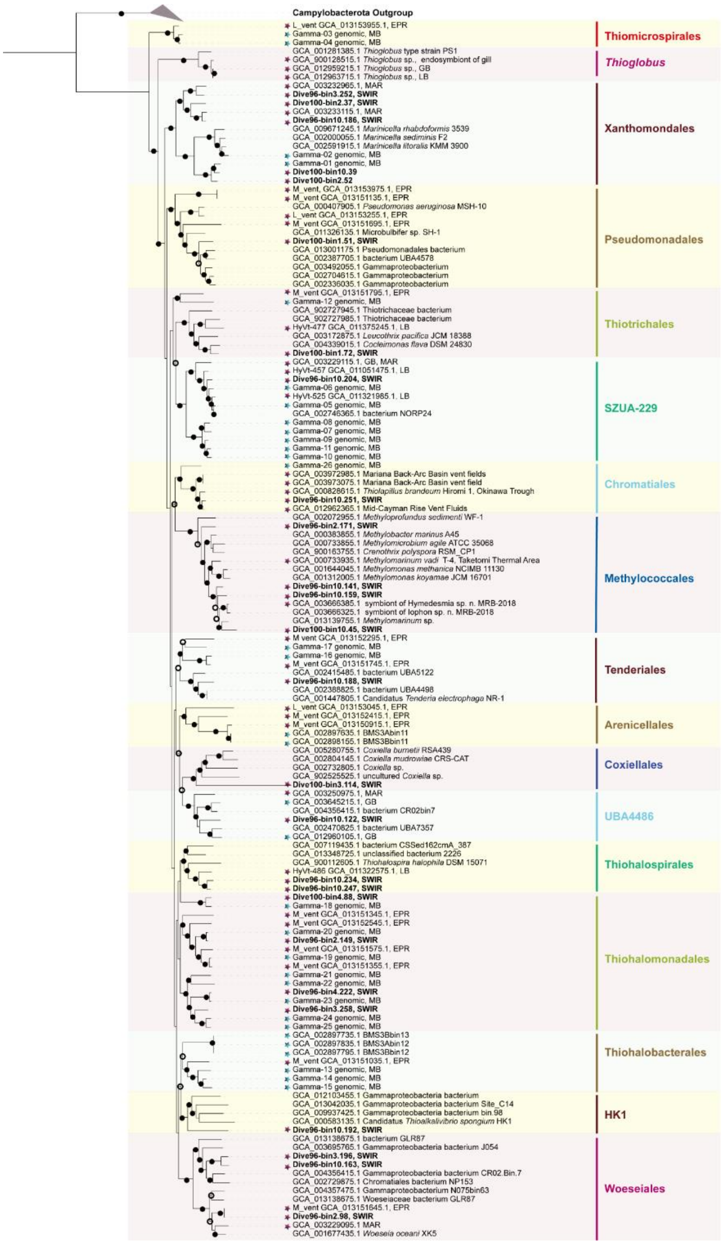

Supplementary Figure 4. Phylogeny of MAGs in Gammaproteobacteria group inferred from concatenated alignment of 16 ribosomal proteins. Red stars marked the genomes recovered from active hydrothermal vents and blue stars marked the genomes recovered from inactive sulfide mineral. For each genome that were recovered hydrothermal vents or inactive sulfide mineral, hydrothermal area where they were detected were pointed out as well. Abbreviations: "MB": "Manus Basin"; "LB": "Lau Basin", "SWIR"; "Southwestern Indian Ridge"; "EPR": "East Pacific Rise"; "MAR": "Mid-Atlantic Ridge". Solid circles marked nodes with bootstrap values not lower than 90. Hollow circles marked nodes with bootstrap values lower than 90 but higher than 70.

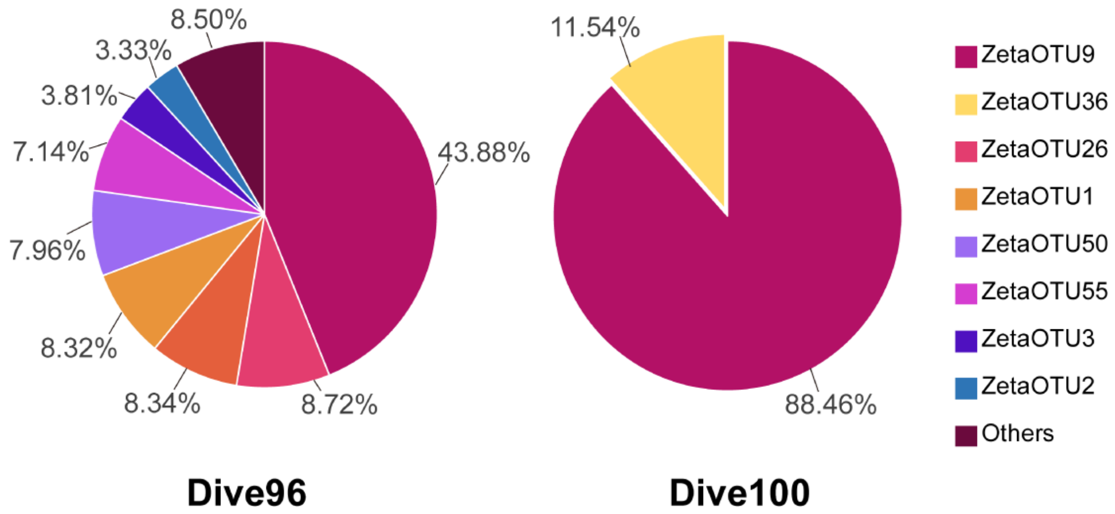

Supplementary Figure 5. ZetaOTU composition of two hydrothermal vent chimneys. To sum up, 20 types of ZetaOTUs identified by ZetaHunter were detected in Dive96 while only ZetaOTU9 and ZetaOTU36 were detected in Dive100.

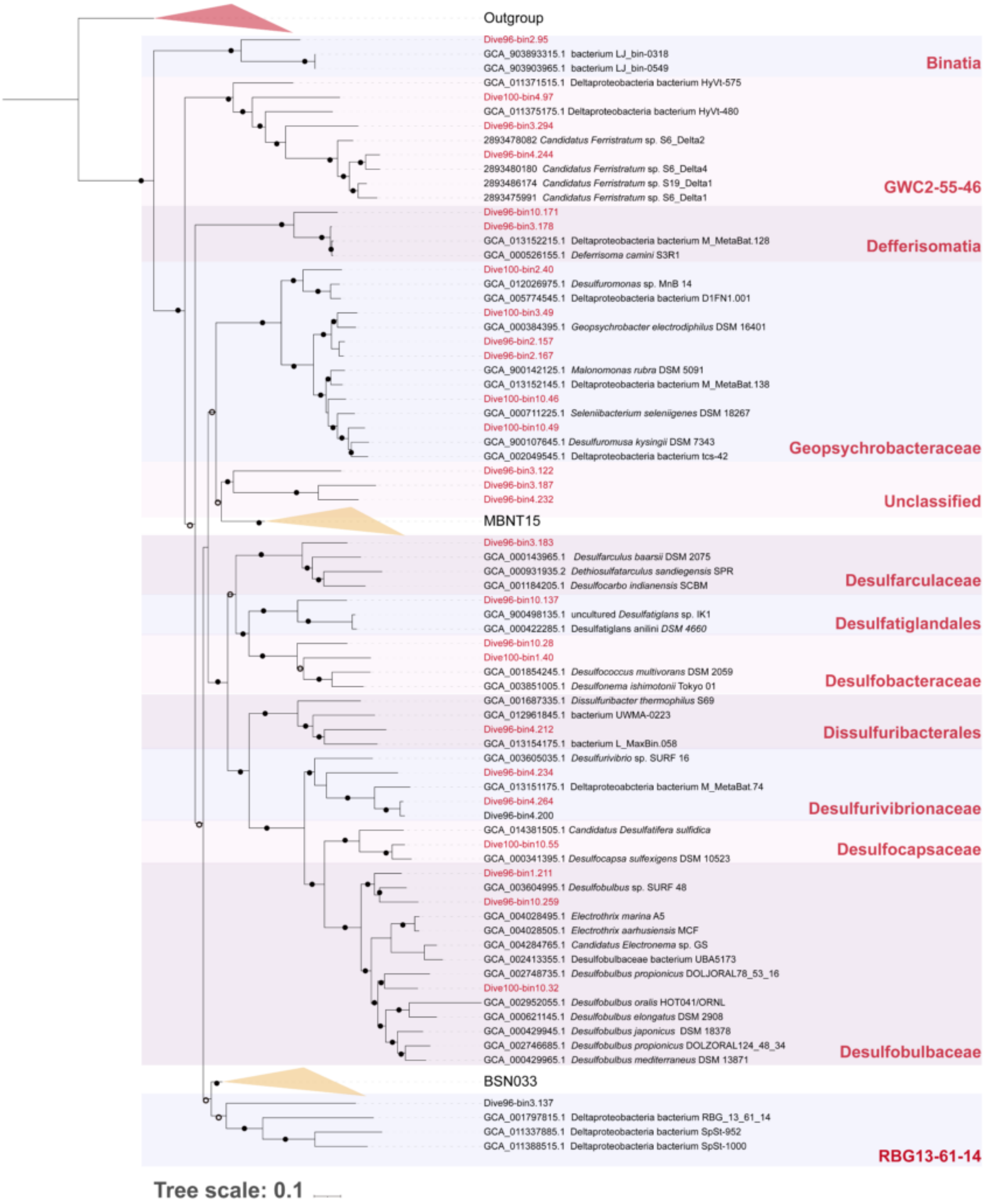

Supplementary Figure 6. Phylogeny of MAGs in Desulfobacterota group inferred from concatenated alignment of 16 ribosomal proteins. Solid circles marked nodes with bootstrap values not lower than 90. Hollow circles marked nodes with bootstrap values lower than 90 but higher than 70.

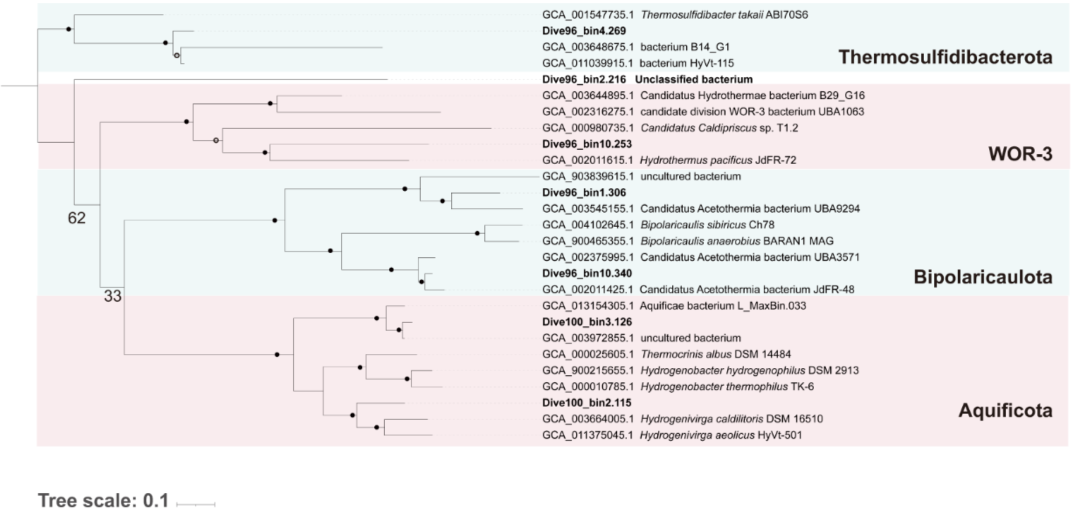

Supplementary Figure 7. Phylogeny of MAGs in Thersulfidibacterota, WOR-3, Bipolaricaulota, Aquificota and unclassified bacterium Dive96_bin2.216 inferred from concatenated alignment of 16 ribosomal proteins. Solid circles marked nodes with bootstrap values not lower than 90. Hollow circles marked nodes with bootstrap values lower than 90 but higher than 70. Taxonomy of Dive96_bin2.216 was undetermined due to weak support from other genomic representatives (bootstrap value lower than 70) in phylogenteic analysis.

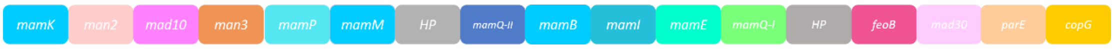

Supplementary Figure 8. Magnetosome gene cluster identified in putative magnetotactic genome Dive96_bin3.242. Abbreviation of "HP" represented hypothetical proteins.

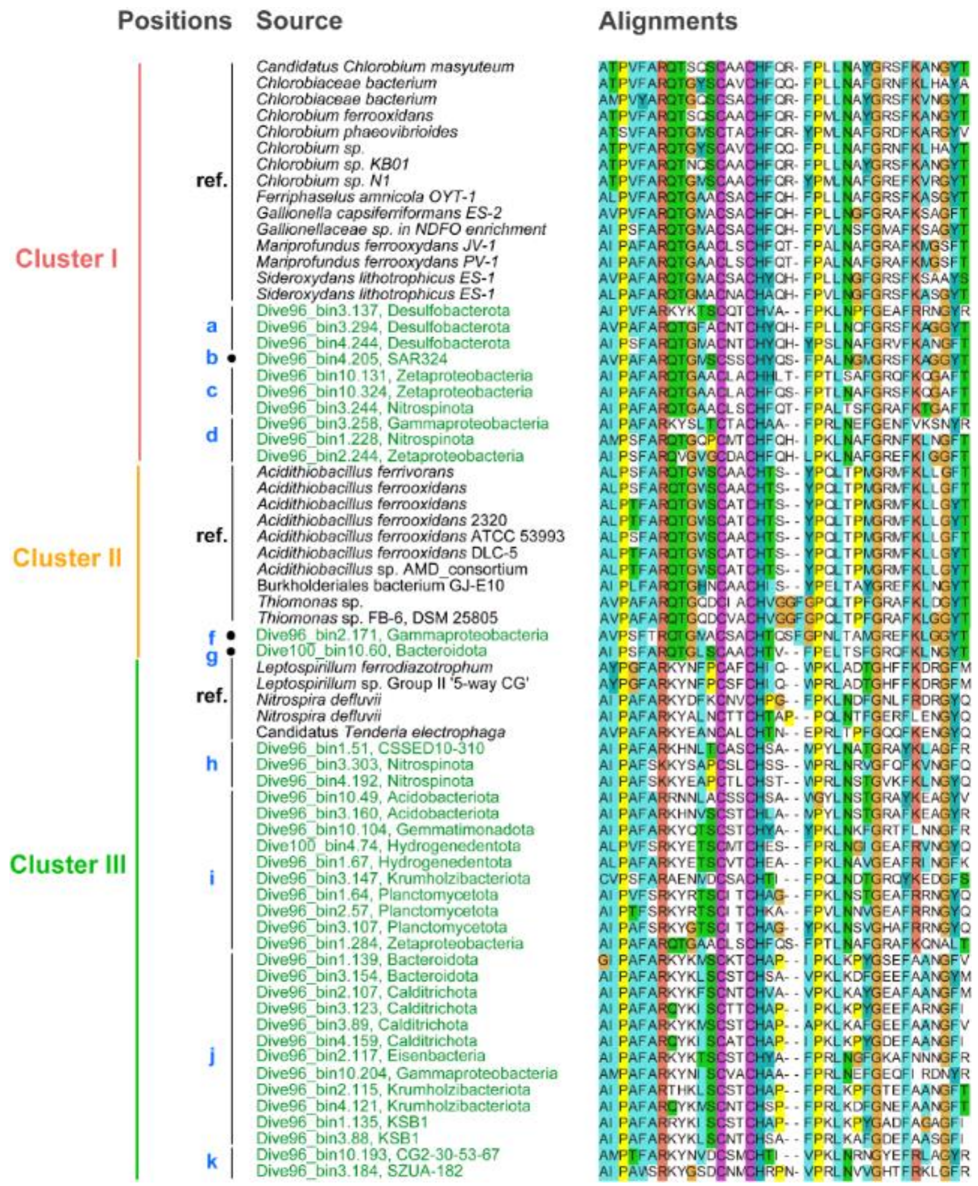

Supplementary Figure 9. Alignment feature at N-terminus CxxCH region of *cyc2* protein sequences identified in FeOBs and *cyc2*-like protein sequences identified in this research. "Source" indicated the organisms where *cyc2* reference sequences were derived or bins and its genome classification where *cyc2*-like sequences were identified in this study. The second coloumn of position indicated the specific positions in phylogenetic tree of *cyc2* protein sequences in Figure 5.

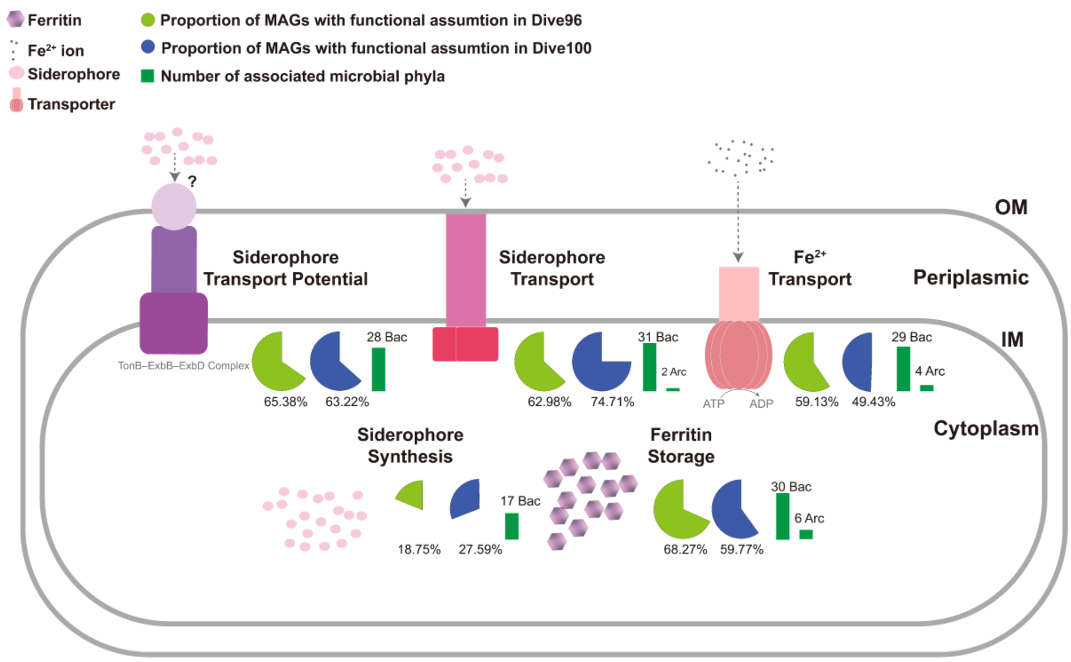

Supplementary Figure 10. Summary of statistics of genomic potential of iron acquisition and storage among 295 MAGs in Dive96 and Divd100. Abbrevations: "OM": "Outer membrane"; "IM": "Inner membrane"; "Bac": "Bacteria"; "Arc": "Archaea".
